# A Novel CpG Island Set Identifies Tissue-Specific Methylation at Developmental Gene Loci

**DOI:** 10.1371/journal.pbio.0060022

**Published:** 2008-01-29

**Authors:** Robert Illingworth, Alastair Kerr, Dina DeSousa, Helle Jørgensen, Peter Ellis, Jim Stalker, David Jackson, Chris Clee, Robert Plumb, Jane Rogers, Sean Humphray, Tony Cox, Cordelia Langford, Adrian Bird

**Affiliations:** 1 Wellcome Trust Centre for Cell Biology, University of Edinburgh, Edinburgh, United Kingdom; 2 Lymphocyte Development Group, MRC Clinical Sciences Centre, Imperial College School of Medicine, London, United Kingdom; 3 Wellcome Trust Sanger Centre, Hinxton, Cambridge, United Kingdom; Genome Institute of Singapore, Singapore

## Abstract

CpG islands (CGIs) are dense clusters of CpG sequences that punctuate the CpG-deficient human genome and associate with many gene promoters. As CGIs also differ from bulk chromosomal DNA by their frequent lack of cytosine methylation, we devised a CGI enrichment method based on nonmethylated CpG affinity chromatography. The resulting library was sequenced to define a novel human blood CGI set that includes many that are not detected by current algorithms. Approximately half of CGIs were associated with annotated gene transcription start sites, the remainder being intra- or intergenic. Using an array representing over 17,000 CGIs, we established that 6%–8% of CGIs are methylated in genomic DNA of human blood, brain, muscle, and spleen. Inter- and intragenic CGIs are preferentially susceptible to methylation. CGIs showing tissue-specific methylation were overrepresented at numerous genetic loci that are essential for development, including *HOX* and *PAX* family members. The findings enable a comprehensive analysis of the roles played by CGI methylation in normal and diseased human tissues.

## Author Summary

The human genome contains about 22,000 genes, each encoding one of the proteins required for human life. A particular cell type (e.g., blood, skin, etc.) expresses a specific subset of protein genes and silences the remainder. To shed light on the mechanisms that cause genes to be activated or shut down, we studied DNA sequences called “CpG islands” (CGIs). These sequences are found at over half of all human genes and can exist in either the active or silent state depending on the presence or absence of methyl groups on the DNA. We devised a method for purifying all CGIs and showed that, unexpectedly, only half occur at the beginning of genes near the promoter, the rest occurring within or between genes. Notably, methylation of CGIs causes stable gene silencing. We tested 17,000 CGIs in four human tissues and found that 6%–8% were methylated in each. Genes whose protein products play an essential role during embryonic development were preferentially methylated, suggesting that gene expression during development could be regulated by CGI methylation.

## Abstract 3

CpG island methylation, an epigenetic phenomenon usually associated with abnormality in disease, is little characterised in the context of "normal" human cells. Here we highlight tissue-specific CpG Island methylation, which frequently associates with developmental genes.

## Introduction

DNA methylation in the mammalian genome arises due to covalent addition of a methyl group to the 5′ position of cytosine in the context of the palindromic dinucleotide, CpG. This modification is established and maintained by a family of DNA methyltransferases that are essential for development and viability [1,2]. The pattern of CpG methylation in the human genome distinguishes two fractions with distinct properties: a major fraction (∼98%), in which CpGs are relatively infrequent (on average 1 per 100 bp) but highly methylated (approximately 80% of all CpG sites), and a minor fraction (<2%) that comprises short stretches of DNA (∼1,000 bp) in which CpG is frequent (∼1 per 10 bp) and methylation-free. The latter are known as CpG islands (CGIs) and they frequently colocalise with the transcription start sites (TSSs) of genes [3,4].

Although CGIs are often free of methylation, there are circumstances in which they become heavily methylated, and this invariably correlates with silencing of any promoter within the CGI. Artificial methylation of CGI promoters has long been known to extinguish transcription when the constructs are introduced into living cells [5]. Moreover, demethylation of endogenous methylated CGIs using DNA methytransferase inhibitors can restore expression of the gene [6]. These findings demonstrate that dense CpG methylation prevents expression of CGI promoters. Because of this biological consequence, it is important to know the extent of CGI methylation in both normal and diseased tissue states. The classical example is X chromosome inactivation in placental mammals, during which hundreds of CGI promoters become methylated and contribute to the stability of gene inactivation on this chromosome [7,8]. Genomic imprinting can also depend upon differential CGI methylation between maternal and paternal alleles [9]. Certain “testis-specific antigen” genes possess CGIs that are methylated in all somatic tissues, but not in testis, where the genes are expressed [10]. Several additional candidates for CGI methylation in normal tissues have been reported [11,12], and the number of cases has recently grown due to large-scale bisulfite sequencing [13] and analysis of promoter methylation using microarrays [14].

In the cases of X chromosome inactivation and genomic imprinting, the biological processes were described initially, and CpG methylation was subsequently implicated through mechanistic studies. To uncover new biological roles for CGI methylation in hitherto undiscovered biological processes, it would be advantageous to comprehensively screen genomic DNA for methylated CGIs in normal or diseased cell types. A persistent limitation affecting this kind of approach has been uncertainty concerning CGI identification [15]. The criteria for designating a sequence as CGI-like are currently exclusively bioinformatic in nature, relying on the differences in the base composition and CpG frequencies (observed/expected) between bulk genomic DNA and CGIs [16,17]. In an attempt to address this limitation and create a resource for future analysis, we developed a method for CGI identification and purification based on their lack of CpG methylation in an otherwise highly methylated genome.

Our method utilised a protein domain with a specific affinity for clustered nonmethylated CpG sites [18,19]. Using this reagent we physically purified DNA sequences that contain clusters of nonmethylated CpG-rich DNA from human blood DNA. Large-scale sequencing of the fraction identified a CGI set that was annotated on the ENSEMBL database. We found that many CGIs in the set were not associated with promoters of annotated genes, but were either within transcription units or between genes. By arraying the intact CGI sequences, we were able to interrogate genomic DNA fractions from several human tissues in order to identify methylated CGIs. The results revealed large numbers of CGIs that are methylated in normal human tissues, many of which showed tissue-specific methylation.

## Results

### A Novel Technique for Purification of CpG Islands

**Figure 1:**
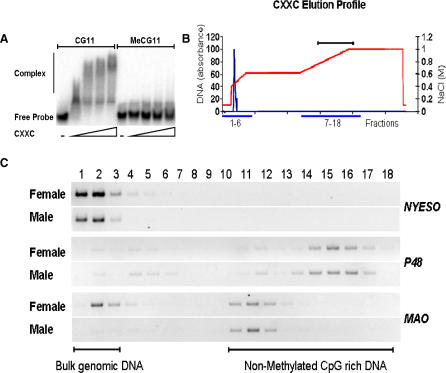
The Immobilised CXXC Domain Specifically Retains DNA Containing Clusters of Nonmethylated CpGs (A) EMSA showing the CXXC complex with a DNA probe containing 27 nonmethylated CpG sites. Nonmethylated probe DNA (CG11) or methylated probe (MeCG11) was incubated with 0, 250, 500, 1,000, or 2,000 ng of recombinant CXXC protein. (B) A typical elution profile of bulk genomic DNA (blue line) from a CXXC affinity chromatography column. Genomic DNA (100 μg) was applied to the CXXC affinity matrix (see Methods) in low salt (0.1 M NaCl) and eluted with a gradient of increasing NaCl (red line; see text). Eighteen fractions were interrogated by PCR (blue lines). The bracket above indicates fractions that were found to contain nonmethylated CGIs. (C) Elution of specific CGI sequences of known methylation status. Methylated CGIs (*NYESO* and *MAO* in females) coelute with bulk genomic DNA (see bracket) whereas nonmethylated CGIs (*P48* and *MAO*) elute at high NaCl concentration.

To enrich for nonmethylated CpG-rich DNA (CpG islands), we developed the technique of CXXC affinity purification (CAP). This uses the cysteine-rich CXXC3 domain that has a high affinity for nonmethylated CpG sites [18,19]. A recombinant CXXC domain from mouse Mbd1[19] was expressed in bacteria, and its binding specificity for nonmethylated CpG sites was confirmed (Figure 1A). The CXXC domain had no detectable affinity for DNA containing only methylated CpGs or for DNA lacking CpGs altogether. We linked the CXXC domain to a sepharose matrix and confirmed that this fractionated DNA fragments according to CpG density and methylation status (unpublished data). All DNA bound to the column at 0.1 M salt. Methylated DNA and CpG-poor DNA eluted at ∼0.4 M NaCl, whereas elution of nonmethylated CpG-rich DNA required 0.6–1.0 M NaCl. To test the behaviour of CGIs on the column, human genomic DNA was digested with MseI (TTAA) [20] and fractionated over the CXXC column (Figure 1B). The reasoning behind use of Mse1 [20] was to cut AT-rich bulk genomic DNA into small fragments (predicted average = 123 bp), but to leave CGIs relatively intact (predicted average = 625 bp). As bulk genomic DNA has a CpG on average every 100 bp, most Mse1 fragments will have too few CpGs to be retained by the CXXC matrix. CGIs on the other hand, with 1 CpG per ∼10 bp, will give rise to long fragments with many CpGs. Eluted fractions were interrogated by PCR using primers specific for a range of known CGIs and non-CGI sequences (Figure 1C). For example, the nonmethylated CGI of the *P48* gene eluted at high salt. The X-linked monoamine oxidase (*MAO*) gene eluted as a single high salt peak from male genomic DNA (where it is nonmethylated), but as two separate peaks at high and low salt when female DNA (with one methylated and one nonmethylated allele) was fractionated. The CGI associated with the *NYESO* testis-specific antigen gene (methylated in somatic tissues) eluted from the CXXC column by low salt as predicted. The data confirm that CAP may be used to purify a CGI fraction from human genomic DNA.

### A Comprehensive CGI Set from Human Blood

**Figure 2:**
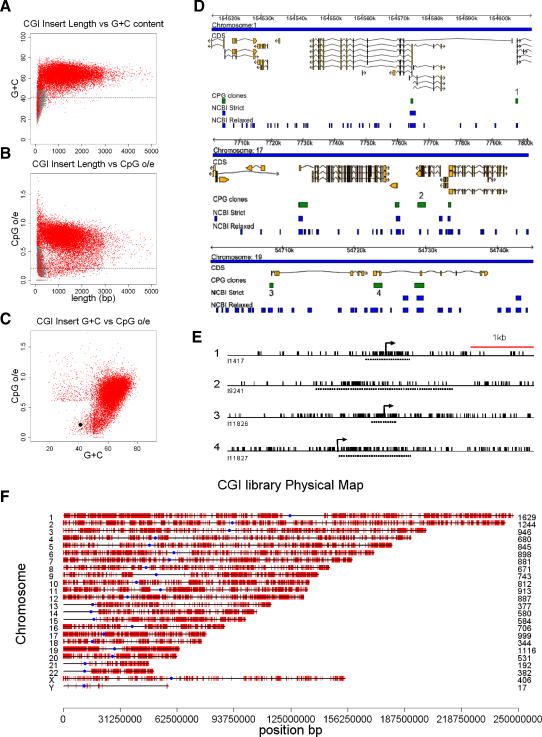
A Library of DNA Sequences that Bind Tightly to the CXXC Column Represents a Comprehensive Set of CGIs (A and B) Plots of fragment length versus G+C content (A) and CpG[o/e] (B) for 28,013 unique Mse1 inserts. Fragments shorter than 512 bp with a G+C content = <50% and a CpG[o/e] = <0.6 (grey dots) were filtered out as contamination. The dashed line indicates the base composition (A) and CpG o/e (B) of bulk genomic DNA. (C) A filtered insert set representing 17,387 CGIs shows a discrete distribution that is distant from bulk genomic DNA (black dot). (D) Three random chromosomal regions showing CGI sequences mapped by ENSEMBL (green bars). Also shown are CGIs predicted by the NCBI-strict and NCBI-relaxed algorithms (blue bars). The directions of transcription of coding sequences (yellow bars) are arrowed. Numbered CGIs (1–4) represent sequences not detected by the NCBI-strict algorithm. (E) CpG maps of the four CGI clones not predicted by NCBI-strict. Transcription start sites in examples 1, 3, and 4 are indicated by arrows. Sequenced MseI fragments are denoted by dashed lines and CpG sites by vertical black strokes. (F) The distribution of cloned CGIs (red strokes) on human chromosomes. The number of CGIs on each chromosome is shown (right) and centromeres are denoted by blue dots.

Most or all CGIs are in a nonmethylated state in sperm, but in addition repetitive elements [21] and telomere-proximal sequences [22], both of which are moderately CpG-rich, are hypomethylated in sperm DNA. To avoid contamination of the CGI fraction with sequences that are nonmethylated, specifically in germ cells, whole human blood was used as a source of CGI fragments. Pooled whole blood DNA from three males was fractionated using the CXXC column. High salt fractions were pooled, diluted, and re-chromatographed before cloning in plasmids. The resulting blood CGI library was analysed by 221,860 sequence reads representing 119,487 genomic templates. These compiled to give 28,013 unique MseI fragments. Plots of DNA insert length versus either G+C content or observed/expected CpG frequency (CpG[o/e]) showed that the great majority of clones exhibited a higher G+C content (average = 62%) and CpG[o/e] (average = 0.71) than bulk genomic DNA (G+C = 41% and CpG[o/e] = 0.2) (Figure 2A and 2B). A fraction of small fragments with sequence characteristics resembling bulk genomic DNA was detected by these plots. As these probably represent contamination, we filtered out fragments shorter than 512 bp that had a GC content less than 50% and/or a CpG[o/e] less than 0.6 (see grey dots in Figure 2A and 2B). The resulting final sequenced set corresponds to 17,387 CGIs and is annotated on the ENSEMBL genome browser (http://www.ensembl.org/index.html. DAS sources: “CPG island clones”). The great majority have classical CGI properties (Figure 2C). Due to their high average GC content, the sequence pass rate was 69%. Assuming that the unsequenced clones reflect the same proportion of CGIs as those that were sequenced, we estimate the total number of CGIs in the library as 25,200. It is likely that a higher proportion of sequence failures affect bona fide CGIs, as GC-richness is known to interfere with sequencing. If so, we estimate that the number of human genomic CGIs may be closer to 30,000.

CGIs are identified bioinformatically as DNA sequences with a base composition greater than 50% G+C and a CpG[o/e] of more than 0.6 [23]. The DNA length over which this condition applies is critical. Initially the threshold most often used was 200 bp, whereas 500 bp is now more commonly applied [17]. These two criteria are formalised as “NCBI-relaxed” and “NCBI-strict,” respectively (http://www.ncbi.nlm.nih.gov/mapview/static/humansearch.html#cpg). The relaxed algorithm predicts 307,193 CGIs in the human genome, which includes many repeated sequences and gene exons. Over 90% of NCBI-relaxed CGIs are not represented in either our library or the set predicted by the NCBI-strict. This and other arguments suggest that the great majority (>90%) are false positives. On the other hand, 77% of clones in the CGI library match CGIs predicted by the “NCBI-strict” algorithm (Table 1). Examples of the coincidence of NCBI-strict predicted CGIs and sequenced CGI clones are illustrated for the three typical regions of the human genome (Figure 2D).

**Table 1:**
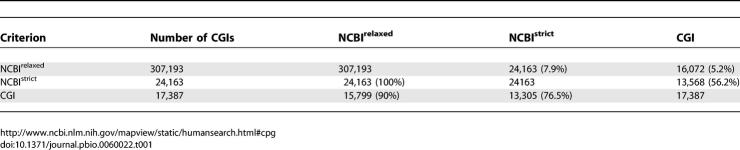
Comparison of Human Blood CGI Set with Bioinformatic Prediction

Altogether, NCBI-strict identifies 24,163 CGIs in the human genome, which accords with the adjusted CGI library estimate of 25,200. The coincidence of these numbers masks significant differences, however, as 23% of CGIs in the library are not detected by the NCBI-strict algorithm (4,082 out of 17,387; Table 1). Four randomly selected examples of library CGIs not detected by NCBI-strict (Figure 2D and 2E, numbered) gave CpG maps resembling CGIs; three of these coincided with the promoters of annotated protein-coding genes (Figure 2D and 2E). The presence of bioinformatically predicted CGIs that are missing from the CGI library is most probably due to sequence failure of ∼31% of library inserts. Analysis of the CGIs missed by the NCBI-strict algorithm shows them to be, as expected, significantly weaker with respect to CpG and G+C content than the total set (Figure S1). It was not obvious, however, that the algorithm could be easily improved based on this information. Relaxation of the sequence parameters reduces the number of false negatives, but leads to increased numbers of false positives. We suggest that CAP identifies islands that fail the NCBI criteria, but reduces the false discovery rate by excluding spurious methylated CpG-rich sequences. Like the majority of CGIs, most NCBI-missed islands are gene-associated, although with an increased incidence of intragenic islands (Table S1). The CGI library therefore includes a significant fraction of bona fide CGIs that are missed by one of the best available algorithms.

CAP defines a set of CGIs that is coherent with respect to clustering of nonmethylated CpG sites. The genomic distribution of these CGI sequences correlates strongly with gene density (Figure 2F). For example, gene-rich Chromosome 19 is also CGI-rich, whereas gene-poor Chromosome 18 is correspondingly CGI-poor. With respect to annotated protein-coding genes, we found that 76% of CGIs are within 1.5 kb of a transcription unit, but only 49% overlap with the TSS (Table 2). It follows that half of CGIs are not TSS-associated, but are either within downstream regions of transcription units (22%) or located in intergenic DNA. Previous studies have detected CGIs at the TSS of 56% of human protein-coding genes [24]. As 43.5% of TSSs overlap sequenced CGIs, we calculate that the sequenced set of 17,387 CGIs represents 78% of the CGI complement. According to this calculation, the total CGI number would be 22,400, somewhat less than the figure of 25,200 deduced from the fraction of sequenced inserts.

**Table 2:**
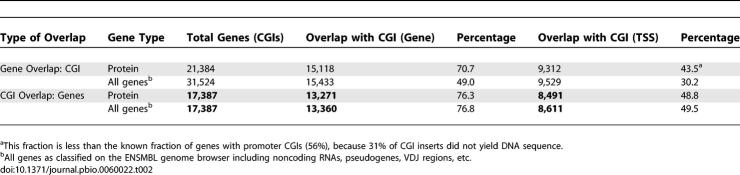
Relationship between CGI Library Inserts and Protein-Coding Genes

### MBD Affinity Purification and Blood CGI Methylation

**Figure 3:**
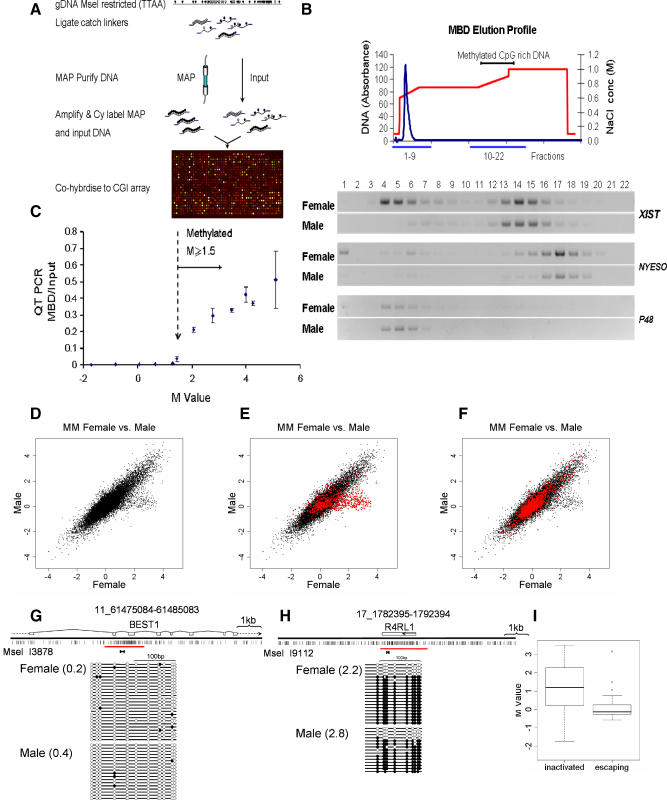
Use of an Arrayed CGI Library to Detect CGI Methylation in Human Blood DNA (A) Schematic showing isolation of densely methylated CGIs using MBD affinity purification based on reference [20]. Open and filled circles represent nonmethylated and methylated CpG sites, respectively. (B) Examples of retention of known methylated CGIs by MBD affinity chromatography. Methylated *XIST* and *NYESO* CGIs elute at high salt concentration, whereas nonmethylated *P48* and female *XIST* co-elute with bulk genomic DNA (blue line) at low salt concentration (red line). (C) *M* values (log_2_[MBD/Input]) >1.5 (dashed vertical arrow) denote DNA fragments enriched by MAP. *M* values are plotted against the ratio of fragment abundance in the MAP probe versus input DNA as determined by quantitative PCR. Error bars represent ± standard deviation. (D–F) MAP CGI array hybridization identifies CGIs that are methylated on the inactive X chromosome. (D) Probes isolated by MAP from male and female whole blood DNA detected female-specific CGI methylation. (E) CGIs on the X chromosome (red dots) often showed female-specific methylation. (F) CGIs on Chromosome 16 (red dots) were indistinguishably methylated between male and female. (G and H) Confirmation of methylated CGIs by bisulfite genomic sequencing. CGI clones I1387 (G) and I9112 (H) are nonmethylated and methylated, respectively, as predicted by the microarray data. Open and filled circles represent nonmethylated and methylated CpG sites, respectively. The genomic locus including annotated transcripts and CpG maps (vertical strokes) are shown above each profile. Each column represents products of amplification by a single primer pair (brackets below CpG map). Each line corresponds to a sequenced DNA strand. Red bars indicate the location of the MseI fragment cloned in the CGI library. (I) The CGI array distinguishes genes inactivated on the X chromosome (inactive) from genes that escape inactivation (escaping). CGIs associated with inactivated genes (*n* = 103) show significantly higher *M* values than CGIs at escaping genes (*n* = 14; KS test: *p* = 1.2 ×10^−5^).

CAP selects CGIs from blood DNA based on their lack of methylation and therefore excludes the small fraction of CGIs (<3%) that are fully methylated in somatic cells from the set [14]. Indeed, CGIs associated with the human testis-specific antigen genes [10], which are methylated in somatic tissues, were not enriched by CAP (Figure 1C) or present in the library (unpublished data). Despite the absence of these fully methylated CGIs, we reasoned that the blood CGI library provides an opportunity to screen for methylation that affects a fraction of all copies of a specific CGI in whole blood DNA. Also, it permits a screen for differential methylation of CGIs in tissues and cell types other than blood. To investigate CGI methylation in normal human tissues, we constructed an array of sequenced CGIs from the library by immobilising single-stranded PCR-amplified inserts on glass slides using 5′-aminolink chemistry as described (http://www.sanger.ac.uk/Projects/Microarrays/arraylab/methods.shtml). As probes for the array, methylated CGIs were enriched from genomic DNA using MBD affinity purification (MAP), which was shown previously to efficiently bind methylated CGIs [20] (Figure 3A and 3B). Human male and female blood DNA was MseI-digested and ligated to universal catch linkers. We verified by PCR that affinity fractionation using MAP effectively separated known methylated CGIs (*XIST* on the active X chromosome and *NYESO*) from bulk genomic DNA and nonmethylated CGIs (*P48* and *XIST* on the inactive X chromosome; see Figure 3B). Male and female DNA fractions were pooled after two rounds of MAP, amplified by linker-mediated PCR, cyanine labeled, and hybridized to the CGI microarray. Quadruplicate hybridisations (inclusive of cyanine dye swaps) gave mean enrichment values (MAP/Input) that allowed a comparison between male and female methylated CGI complements. As expected, these were positively correlated (*R* = 0.865 Pearson correlation) suggesting similar overall patterns. As the library comprises MseI fragments that sometimes overlap minimally with the cognate CpG-rich region, we chose to disregard data from spots that contained DNA with an average CpG frequency (observed/expected) of less than 0.5. Although the omitted fragments often denote CGIs, they include too little of the CpG-rich domain to be reliable for detection of MAP probes. This refinement reduced the number of analysable CGIs on the array to 14,318. To assess the relationship between hybridization signal relative to input and degree of enrichment by MAP, we measured a selection of CGIs in the probe by quantitative PCR and compared this data with the *M* values (log2 [MAP signal]/[Input signal]) for those sequences (Figure 3C). The results established that *M* values greater than 1.5 denote CGIs that are significantly enriched by MAP and therefore methylated. CGIs of the *BEST1* and *R4RL1* genes were predicted to be nonmethylated (*M* = 0.2–0.4) and methylated (*M* = 2.2–2.8), respectively, based on the array data. Bisulfite genomic sequencing confirmed this expectation (Figure 3G and 3H).

The major difference in CGI methylation between male and female DNA was expected to be due to X chromosome inactivation (see also [25]). We therefore compared the methylation status of CGIs on Chr 16 and Chr X in male versus female DNA. Chr 16 CGIs did not vary between males and females, whereas Chr X CGIs were significantly enriched in female DNA as predicted (Figure 3D–3F; Table S2). Studies of human X chromosome inactivation have indicated that a proportion of genes escape inactivation and are therefore expressed from both chromosomes [26,27]. By comparing the microarray data for a set of inactivated and escaping CGIs, we found that inactivated genes had significantly higher *M* values (*p*-value = 1.213 ×10^−5^) (Figure 3I). This finding affirms the long-standing link between CGI methylation and gene silencing and validates the present experimental system as a means of detecting genes that are shut down in this way.

### Differential CGI Methylation in Human Tissues

**Figure 4:**
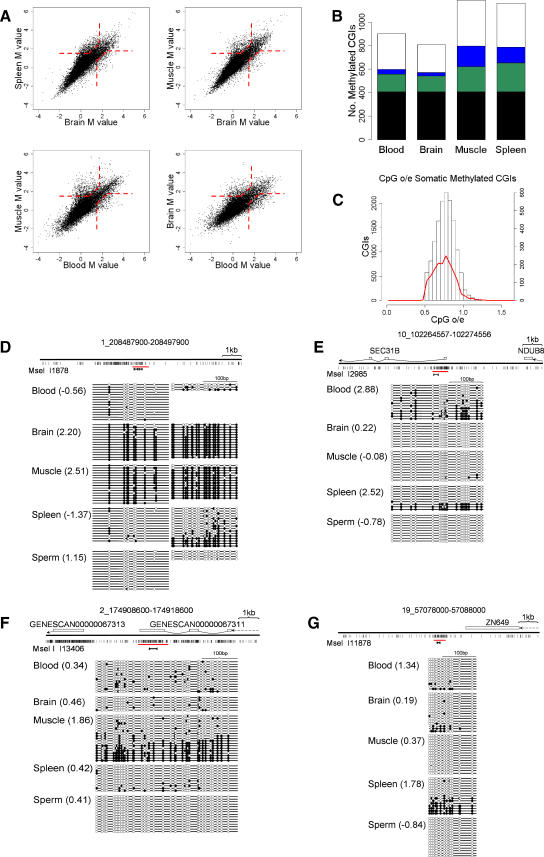
Tissue-Specific CGI Methylation in a Panel of Human Tissues (A) Examples of pairwise comparisons using MAP CGI probes derived from blood, brain, muscle, and spleen. Broken red lines indicate threshold *M* values used to determine differential CGI methylation. (B) Frequencies of methylated CGIs in blood, brain, muscle, and spleen. The following catagories are represented: CGIs methylated in all tested tissues (black); CGIs methylated in more than one tissue tested but not all (green); CGIs methylated in one tissue only (blue); CGIs methylated in one tissue tested but unclassified in other tissues (white). (C) Somatically methylated CGIs display a very small but significant reduction in CpG[o/e] (0.75) relative to the whole CGI set (0.77; *n* = 1,657 and 12,661, Wilcoxon rank test: *p*-value: 1.022e^−11^). The histogram shows the CpG[o/e] profile for the total CGI set (white bars) overlaid with the CpG[o/e] profile for methylated CGIs (red line). (D–G) Confirmation of candidate CGIs showing evidence of tissue specific methylation by bisulfite genomic sequencing. Layout is as for Figure 3G.

Methylation of CGIs on the inactive X chromosome and at imprinted genes is well known, but CGI methylation at other chromosomal loci in normal cells and tissues is incompletely characterized [12,13,28,29,30]. To investigate this issue on a large scale, we probed CGI arrays with MAP fractions from genomic DNA (three individuals per pool) of brain, muscle, spleen, and sperm in addition to blood (Figure 4A). MAP enrichment of methylated CGIs in sperm DNA consistently failed to generate enough DNA for labeling using our standard PCR amplification conditions and was therefore not analysed further. We conclude that the level of CGI methylation in sperm is far lower than in any of the somatic tissues. Taking *M* values greater than 1.5 to signify methylation, we observed between 5.7% and 8.3% of CGIs methylated in the somatic tissues that were tested (Figure 4B; Table 3; Dataset S1). Some CGIs were methylated in common between all the tested somatic tissues, whereas others were methylated in only one or a subset of the tissues. We noted that methylated CGIs disproportionately involved those that are remote from the TSS of an annotated gene. In the dataset as a whole, only 8% of TSS CGIs showed evidence of methylation in at least one tissue, whereas 22% of 3′ CGIs were methylated (Table 4). Do the methylated CGIs differ in sequence characteristics from CGIs that remain methylation-free? We plotted the CpG[o/e] frequencies of 1,657 CGIs that acquired methylation in one or more tissues and found a mean CpG[o/e] of 0.77 compared with 0.75 for methylated CGIs (Figure 4C). Though statistically significant (*p*-value = 1.413e-10) the biological significance of this small difference is unclear.

**Table 3:**
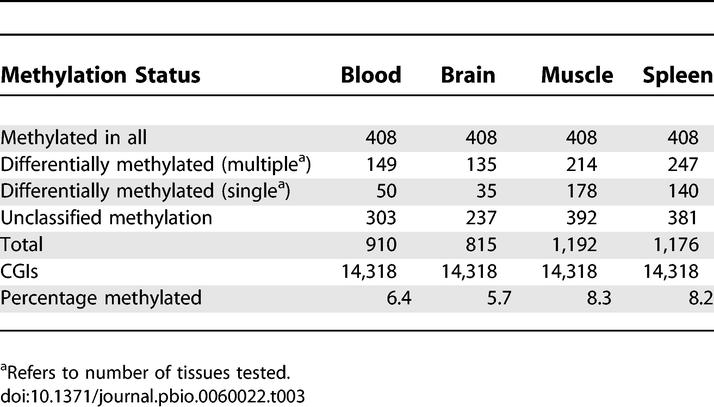
CGI Methylation in Human Tissues

**Table 4:**
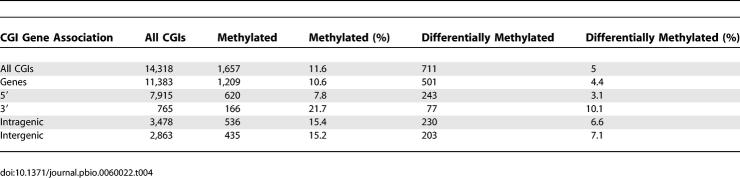
Location of Methylated CGI Relative to Protein-Coding Genes

**Figure 5:**
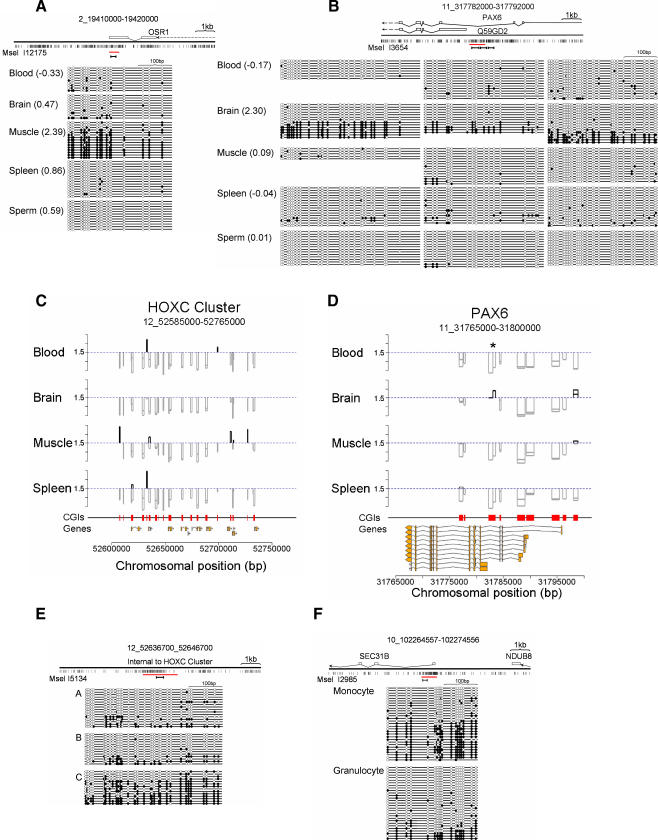
Tissue, Cell-Type, and Individual-Specific CGI Methylation at Developmental Gene Loci (A–B and E–F) Bisulfite genomic sequencing confirmed tissue-specific CGI methylation associated with the developmental genes *OSR1* (A) and *PAX6* (B). Multiple CGIs (red boxes) span the *HOXC* (C) and *PAX6* (D) gene loci. Plots of the MAP-CGI array profiles for blood, brain, muscle, and spleen identify tissue-specific CGI methylation (vertical black bars extending above *M* = 1.5). Gray bars extending downwards below *M* = 1.5 (broken blue line) represent nonmethylated CGIs. The region of *PAX6* analysed by bisulfite genomic sequencing (see Figure 5B) is indicated (asterisk in panel D). Tick marks on the *y*-axis are spaced at intervals of 1 *M* value unit. Coding sequences are diagrammed as yellow bars. (E) Individual-specific CGI methylation internal to the *HOXC* cluster in muscle DNA. (F) Cell type–specific methylation is seen at the *SEC31B* promoter CGI in monocytes and granulocytes derived from whole human blood. Bisulfite genomic sequencing results (A–B and E–F) are diagrammed as in Figure 3G.

We checked by bisulfite sequence analysis a panel of seven CGIs with *M* values suggestive of tissue-specific methylation (*M* values differing between tissues by >0.75). In each case, bisulfite data confirmed the microarray predictions. CGI I1878 is not associated with an annotated gene (±1.5 kb) and is methylated exclusively in muscle and brain (Figure 4D). CGI I2985 spans the transcription start site of the *SEC31B* gene, whose product is implicated in vesicular trafficking, and is compositely methylated only in blood and spleen (Figure 4E). CGIs I13406 (Figure 4F) and I12175 (Figure 5A) are methylated specifically in muscle. These overlap the predicted gene 67313 and the 3′ end of *OSR1*. CGI I3654, which is associated with the promoter region of an annotated *PAX6* transcript (Q59GD2), previously shown to contain methylated CpG sites [31], is specifically methylated in brain (Figure 5B). I11878 is a 3′ CGI of *ZN649* and is only methylated in spleen (Figure 4G).

Many methylated CGIs were associated with genes that are essential for development (Figure 5). This was confirmed by analysis of gene ontology, which showed significant overrepresentation of genes whose products are involved in developmental processes, including ectoderm and mesoderm development, neurogenesis, and segment specification (Table S3). Transcription factors, including homeobox family members and other DNA binding proteins, were twice as abundant as expected by chance. Other gene categories did not show significant enrichment. Among the CGIs whose methylation status was confirmed by bisulfite sequencing, *PAX6* is involved in eye development and neurogenesis [32], the *HOXC* cluster lays down the embryonic body plan, and *OSR1* is related to a gene involved in *Drosophila* gut development. We examined the extended *HOXC* and *PAX6* loci for CGI methylation status using the MAP-CGI array data. Our library identified 19 CGIs within the 150-kb *HOXC* gene cluster of which eight were methylated differentially in blood, muscle, and spleen (Figure 5C). Brain was the only tissue that lacked obvious *HOXC* CGI methylation. Of nine CGIs near *PAX6*, two showed differential methylation. In addition to brain-specific methylation of the *PAX6-Q59GD2* CGI (see Figure 5B), we observed methylation of a CGI upstream of the major *PAX6* promoter in muscle and brain (Figure 5D).

The majority of CGIs identified as methylated by MAP-CGI array hybridization display composite methylation (Figures 3, 4, and 5), whereby DNA strands at a specific locus were either heavily methylated or essentially nonmethylated. This can explain why CGIs that were initially selected by being nonmethylated in blood DNA (by CAP) nevertheless register as methylated by MAP-CGI array analysis. One potential explanation for composite CGI methylation is that different individuals within the tissue pools exhibit different CGI methylation. To look for such “polymorphism,” we examined CGI I5134, which is within the *HOXC* cluster and shows composite methylation by bisulfite genomic sequencing. Analysis of individuals by MAP-CGI arrays showed highly significant differences between individual C and individuals A and B (Figure 5E). This strikingly confirms individual variability in methylation at this CGI.

Another potential explanation for composite CGI methylation is that cell types within the tissue sample possess different CGI methylation profiles. Blood, for example, consists of monocytes and granulocytes, each of which is subdivided into other cell types. As CGI I2985 was methylated at about half of DNA strands in blood, we tested the level of CGI methylation in DNA from monocytes and granulocytes separately. The results showed that monocytes had high methylation levels at this CGI, whereas granulocytes had very low methylation (Figure 5F). These findings indicate a developmental origin for cell type–specific methylation at this genomic CGI.

## Discussion

### A Comprehensive CGI Set

We describe the characterisation of a comprehensive, verified CGI set derived from human blood genomic DNA that will be beneficial for studies of CGIs in normal human tissues and in disease settings. By focusing on CGIs alone, we excluded ∼98% of the genome from our analysis. While it will ultimately be important to know in detail the methylation status of whole genomes, this currently represents a technical challenge that has been addressed only for the small-genomed plant *Arabidopsis* [33,34]. These studies used indirect microarray-based methods for mapping DNA methylation that depend upon probes enriched in methylated domains. Current enrichment methods require clusters of CpG methylation, which are notably absent from the CpG-deficient majority of the mammalian genome. As a result, much bulk genomic DNA is beyond the resolution limit of this approach. Whole genome bisulfite sequencing, the most direct and reliable method for mapping methylated sites, has not yet been attempted in any organism. We therefore decided to study a discrete genomic fraction with evident biological relevance whose methylation status can be interrogated using microarray-based methods.

To isolate nonmethylated duplex CGIs from total genomic DNA, we harnessed the binding specificity of the CXXC protein domain. Extensive sequencing of the resulting library confirmed that CGIs represent a discrete fraction of the human genome with shared DNA sequence characteristics. The present CGI set supercedes a previous human CGI library that was prepared in our laboratory using an indirect affinity purification procedure [20]. The initial library was not comprehensive and appears to have acquired significant levels of non-CGI contamination following amplifications. We estimate that the new library represents ∼25,000 CGIs, of which ∼60% have been arrayed as full-length single strands on glass slides. Additional analysis of inserts that initially failed conventional sequencing strategies will generate an array that covers the great majority of CGIs that are nonmethylated in human blood. The choice of blood DNA as a starting material necessarily excludes from the set any CGIs that are nonmethylated in germ cells, but densely methylated in the soma [14]. In the future, it will be instructive to compare an exhaustive sequence analysis of this set with comparable sequences isolated by CAP from sperm DNA.

The library prepared using CAP defines CGIs based on the empirical criterion of clustered nonmethylated CpGs, whereas criteria based purely on base sequence and composition necessarily ignore methylation status. Comparing our set with predicted CGIs on the NCBI database shows good overlap with predictions based on the “strict” algorithm. The CGI library did, however, identify 23% of CGIs that were negative by this criterion. This suggests that the software for DNA sequence-based CGI identification misses almost one in four CGIs that the more biological criterion of CAP is able to include. Recent CGI analyses identified large numbers of human CGI promoters that are enriched in methylation at lysine 4 of histone H3, a mark of transcriptional activity [14,35,36]. Since it has been proposed that hypomethylation is dependent on germ line and early embryonic transcription [3], we determined the overlap between our CGI set and the H3K4 sites in human embryonic stem cells [37]. We calculate that 90% of CGIs in the filtered set (14,318) coincide with H3K4 methylated promoters that were reported in the chromatin study. A better test of the relationship between CGIs and H3K4 methylation islands in ES cells is to exclude promoters of annotated genes and focus on intra- and intergenic CGIs. Here again, a high proportion (75%) of CGIs overlap with H3K4 methylation islands. These findings are compatible with the notion that the presence of CGIs is connected with specialised chromatin configurations in early embryonic cells. An intriguing proposal is that H3K4 methylation may be incompatible with docking of de novo methyltransferases [38]. This could in theory insure that these regions remain free of CpG methylation at a time when the rest of the embryonic genome is subject to global methylation.

We found that 49% of CGIs overlap the TSS of an annotated gene. In considering the function of the half of CGIs that are remote from an annotated TSS, it is noteworthy that several intragenic CGIs have been shown to coincide with previously unforeseen promoters that initiate bona fide transcripts [39,40]. This raises the possibility that all CGIs function as promoters and are therefore TSS-associated [40]. In this connection, it is of interest that genome-wide analysis by tiling arrays detected over 10,000 unanticipated human transcripts, many of which may represent noncoding RNAs [41]. It is conceivable that many inter- and intragenic CGIs mark promoters that drive the synthesis of these novel transcripts. The noncoding transcripts *XIST* and *AIR*, for example, whose RNA products play regulatory roles [42–44], both initiate within CGI promoters. The proximity of many methylated CGIs to developmentally important genes raises the possibility that putative CGI transcripts play regulatory roles during development. Recent analyses of the human *HOX* gene cluster highlight the functional importance of noncoding RNAs [45]. Large numbers of potential CGI promoters within *HOX* gene loci may therefore contribute to the regulation of these complex loci.

### CGI Methylation in Normal Tissues

CGI methylation has been extensively studied in cancers and their derivative cell lines, but relatively less attention has been paid to the phenomenon in normal tissues. Several studies have reported somatic CGI methylation, but in early examples the bioinformatics procedure used to identify these sequences was often equivalent to the NCBI-relaxed algorithm, which generates a large excess of questionable CGI candidates. The *MASPIN* gene, for example, scores as a methylated CGI promoter by the relaxed criterion [28], but it is not detected as such either by the NCBI-strict algorithm or by CAP (unpublished data). A recent report addressing the methylation status of 16,000 human promoters identified that 3% of TSS-associated CGIs are normally methylated in somatic tissue [14], which is somewhat below the levels observed in our study (7.8%; Table 4). We detect a much higher frequency of methylation at nonpromoter CGIs (average = 16%), which are obviously absent from promoter arrays. In particular, 22% of CGIs near the 3′ ends of genes are methylated. Extensive bisulfite sequence analysis [13] surveyed 512 CGIs on Chrs 6, 20, and 21 and reported 9.2% to be methylated in somatic tissues. This is similar to the overall level of 11.6% methylation among 14,318 CGIs detected by our study (Table 4).

Our findings raise important questions about the relationship of CGI methylation to gene expression. On the X chromosome, it is clear that methylated CGIs correlate with inactivated genes whereas unmethylated CGIs correlate with genes known to escape inactivation. The generalisation that CGI methylation silences promoters is therefore supported (see also [25]). The relevance to gene expression of the autosomal methylated CGIs identified here is complicated by the frequent presence of both methylated and nonmethylated alleles in a specific tissue (see below). This means that even if CGI methylation silences a promoter completely, large changes in gene expression are not to be expected. Also, many CGIs are not at promoters of annotated genes, but are within or between transcription units. Their function with respect to transcription, if any, may be positive or negative. Finally, any transcripts originating from these “orphan” CGIs have yet to be identified and cannot be tested. For these reasons, it is difficult to make predictions about the effect of CGI methylation on global transcription levels. We nevertheless mined published expression microarray data to determine whether tissues in which a specific set of promoter CGIs was methylated expressed the associated genes at a different level from tissues where the same CGI was unmethylated. The results showed no obvious correlation between CGI methylation and expression. This, therefore, remains an open question that demands detailed analysis of specific cases.

Genes that play an important role in development were prominent among the set of methylated CGIs identified by MAP-CGI array hybridization. Out of 109 CGI-associated genes that contain homeobox-like domains, 27 (∼25%) were unmethylated in at least one tissue compared with ∼11% of all CGI-associated genes (see Table 4). Specifically, we identified 79 CGIs in the four human *HOX* gene clusters A–D, of which 22 were methylated in at least one of the tissues that we tested. Given the relatively small selection of tissues analysed in the study, the actual frequency of *HOX* CGI methylation in all human tissues is likely to be higher than one in four. Interestingly, methylation of *HOX* gene CGIs is also reported in cancers [46], raising the possibility that cancer CGI methylation patterns mimic patterns that arise during development. A potential link between normal development and cancer is suggested by the finding that CGIs methylated in cancer preferentially include promoters that are marked by association with polycomb group proteins in embryonic stem cells [47–49]. In contrast, we found little difference between the fractions of all CGIs (5.9% = 845/14,318) and of methylated CGIs (7.7% = 127/1,657) that were polycomb-associated in embryonic cells [37]. The origins of CGI methylation in cancer may be distinct from the mechanisms that lead to CGI methylation in normal tissues.

It was reported that the most CpG-rich CGIs among 512 analysed on Chr 6, Chr 20, and Chr 22 were never methylated, suggesting that the CpG-richness may protect from methylation [13]. In a larger CGI set, we detected a very small, but statistically significant, difference in sequence properties between CGIs that become methylated and those that remain immune in the tested cell types. The mean CpG[o/e] was 0.75 for methylated CGIs compared with 0.77 for bulk CGIs (Figure 4C). Bock and colleagues [50] identified sequence features that were predictive for CGI methylation, including specific repeats, sequence patterns, and DNA structure. Contrary to predictions of this method, methylated CGIs were significantly depleted in repetitive elements and showed no difference in predicted base twist. We did, however, observe small, but statistically significant, increases in simple sequence elements (TGTG/CACA) and base-stacking energy (see Figure S2). The biological relevance of these minimal differences is uncertain.

Weber and coworkers [14] identified ∼2,000 promoters out of 16,000 that were more susceptible to methylation than CGIs themselves. These so-called “weak CpG islands” had an average CpG[o/e] ratio intermediate between CGIs and bulk genomic DNA. We have determined that 75% of weak CpG islands reported by Weber et al. are absent from the CGI library. Weak CGIs may be depleted because they are heavily methylated and therefore not enriched by CAP. Indeed, 22 methylated weak CpG islands [14] were not detected in our library. Alternatively, their relatively low CpG density and somewhat elevated frequency of Mse1 sites may result in too few CpGs per fragment for efficient retention by the CXXC matrix.

### Composite Methylation of CGIs

Those CGIs that were methylated often showed a mixture of heavily methylated and nonmethylated strands by bisulfite analysis. There are several possible explanations for composite methylation patterns. Firstly, at the highest level, it is possible that different individuals contributing to the DNA pool are polymorphic with respect to this epigenetic mark. We analysed specific CGIs in muscle DNA from three individuals and found evidence of individual variation of this kind. A large-scale survey would be required to determine the extent of inter-individual variability. A second possibility is that cells within the analysed tissue are heterogeneous with respect to CGI methylation. Each of the analysed tissues consists of multiple differentiated cell types that should be analysed separately to address this possibility. Analysis of three compositely methylated CGIs in blood showed one that was highly methylated in monocytes, but weakly methylated in granulocytes, indicating that cell type–specific CGI methylation underlay heterogeneous DNA methylation. A third possible explanation for composite methylation is monoallelic methylation. A previous study of 149 CGIs on Chr 21q detected three that were mono-allelically methylated, indicating that this explanation also accounts for some cases of composite CGI methylation [12].

## Methods

### Plasmid cloning and recombinant protein purification.

Cloning of the His-CXXC construct from murine *Mbd1a* was described previously [19]. The MBD construct was subcloned from pET30bhMeCP2 [51]. A fragment of human MeCP2 corresponding to amino acids 76–167 was PCR-amplified and ligated into the Nde1 and EcoR1 sites of pet30b (Novagen) to generate a C terminally His-tagged pet30bMeCP2_76–167. Primers: pet30bMeCP2_76–167Nde1 CGG TTC ATA ACC ATA TGG CTT CTG CCT CCC CCA AAC AGC GG and pet30bMeCP2_76–167EcoR1 CGG AAG TCA AAG AAT TCT CAT CAG TGG TGG TGG TGG TGC CGG GA. Recombinant peptides were purified from 10 l of induced BL21(DE3)pLysS (Stratagene) culture on Nickel Charged Fast Flow Chelating Sepharose (GE Healthcare). The CXXC construct was further purified by cation exchange using Sp-Sepharose (GE Healthcare) cation exchange as previously described [51]. Recombinant protein was bound to Nickel sepharose prior to longer term storage.

### Electrophoretic mobility shift assay (EMSA).

CXXC-EMSA was carried out essentially as described in [19]. Briefly, binding reactions including 0, 250, 500, 1,000, or 2,000 ng of purified recombinant His-CXXC were preincubated in 1× binding buffer (5 × binding buffer: 30 mM Tris-HCl [pH8], 750 mM NaCl, 5 mM DTT, 30 mM MgCl_2_, 15% Glycerol, 50 ng/μl BSA, and 0.05 μg/μl of poly(dAdT) (Amersham). End-labeled CG11[52] probe (1 ng) was added to each reaction and incubated for a further 25 min. Complexes between probe DNA and the CXXC domain were resolved on a 1.3% agarose Tris-borate-EDTA gel and imaged by Phosphor Imager (Molecular Devices).

### Human DNA samples.

Whole blood was collected from voluntary donors and used in anonymized pools. Donors were aware of, and consented to, its use for preparation of DNA. Monocyte and granulocyte cells were prepared from whole human blood using Ficoll gradient centrifugation. Whole blood (3 ml) was layered onto an equivalent volume of Histopaque-1077 Ficoll (Sigma-Aldrich) and sedimented according to the manufacturer's instructions. Mononucleocytes were recovered from the plasma-ficoll interphase and granulocytes from the cell pellet. Whole human blood, monocyte, and granulocyte DNA was extracted using the Genomic-tip 500/G (Qiagen 10262) genomic DNA purification kit. Sperm DNA was prepared as described [53]. Human skeletal muscle, spleen, and brain genomic DNAs were purchased from Ambion.

### CXXC affinity purification.

50–60 mg of recombinant CXXC was dialysed into W1 buffer (50 mM sodium phosphate buffer [pH8], 300 mM NaCl, 10% glycerol, 15 mM ß-mercaptoethanol, 0.5 mM PMSF), bound to nickel-charged sepharose, and then washed with 10 column volumes (CVs) of W1, 10 CVs of W2 (W1 + 10 mM Imadazole), and 10 CVs of W1. Beads were packed onto a 1-ml Tricorn chromatography column (GE Healthcare). Mse1 digested male DNA (100 μg) pooled from three individuals was bound to the CXXC column in 90% CA buffer (20 mM Hepes [pH7.9], 0.1% Triton X-100, 10% glycerol, 0.5 mM PMSF, 10 mM 2-Mercaptoethanol) and 10% CB buffer (CA + 1 M NaCl). Equilibrated DNA was then eluted over an increasing NaCl gradient of 10%–100% CB buffer (Figure 1B). Fractions (3 ml) were collected and 200 μl of each was precipitated and resuspended in 40 μl 1 × TE buffer. Aliquots were PCR- interrogated using Redhot taq DNA polymerase (Abgene) for *XIST* (for CACGTGACAAAAGCCATG, rev GGTTAGCATGGTGGTGGAC), *NYESO* (for CCCAGCGTCTGGTAACCATC, revCCACGGGACAGGTACCTC ), *MAO* (for CGGGTATCAGATTGAAACAT, rev CTCTAAGCATGGCTACACTACA), *P48* (for cagaaggtcatcatctgcca, rev tgagttgtttttcatcagtcca) under the following conditions: 2 min at 94 °C; followed by 30 cycles of 94 °C for 50 s, T^ann^ °C for 50 s, 72 °C for 1 min; and a final extension of 72 °C for 7 min. PCR products were resolved on a 1.5% TAE-agarose gel (Figure 1B). Fractions retaining nonmethylated CpG-rich Mse1 fragments (Figure 1B) were pooled, diluted with CA buffer, and re-chromatographed. The relevant fractions were precipitated and ligated into the NdeI site of pGEM5zf- (Promega).

### CGI library sequencing.

The clone set was arrayed into 384-well plates in glycerol for long-term storage. Copies were taken and DNA prepared for sequencing using a modified alkaline lysis method. Cells were lysed in glucose, Tris, EDTA (pH 8) buffer plus NaOH and SDS and spun through Millipore Montage filter plates directly into propan-2-ol to precipitate, followed by elution in water. In all, 172,800 clones were sequenced forward and reverse using T7 and SP6 primers and BigDye V3.1 chemistry, under the following conditions: 30 s at 96 °C, followed by 44 cycles of 92 °C 8 s, 55 °C 8 s, 60 °C 2 min. Samples were separated using 3730 XL sequencers (Applied Biosystems). Extraction was performed using sequence analysis v3.1, and base-called using Phred [54]. DNA sequences were identified using NCBI36 and mapped using the ENSMBL Genome Browser (http://www.ensembl.org/index.html). CGIs that mapped within 1.5 kb of annotated genes were considered to be gene-associated in order to take into account mis-annotation of transcription start sites within poorly defined 5′ UTRs.

### Microarray fabrication.

Amino-linked clone insert amplicons were generated by vector-specific PCR in 50 mM KCl, 5 mM Tris (pH 8.5), and 2.5 mM MgCl_2_ including 1 M Betaine (10 min at 95 °C; followed by 35 cycles of 95 °C for 1 min, 60 °C for 15 s, 72 °C for 7 min; and a final extension of 92 °C for 10 min; 5′ aminolink forward primer 5′ - CTC ACT ATA ggg CgA ATTg g −3′ reverse primer 5′ -CgC CAA gCT ATT TAg gTg AC-3′). PCR products were ethanol-precipitated and resuspended in 1 × microarray spotting buffer (250 mM sodium phosphate [pH 8.5], 0.01% sarkosyl, 0.1% sodium azide). Arrays were spotted onto amine-binding slides at 20–25 °C, 40%–50% relative humidity. After an overnight incubation in a humid chamber, the slides were blocked (1% ammonium hydroxide for 5 min, followed by 0.1% SDS for 5 min) and denatured (95 °C ddH_2_O for 2 min), rinsed in ddH_2_O, and dried by centrifugation for 5 min at 250 ×*g*.

### MAP, labeling, and microarray hybridization.

Human tissue DNA pooled from three individuals was digested with Mse1, phosphatase-treated, and ligated to 5 μmol of phosphorylated catch-linkers (upper_GGT CCA TCC AAC CGA TCT and lower_CCA GGT AGG TTG GCT AGA AT phosphate) that had been annealed in 1× TE for 5 h. DNA was bound to an MBD chromatography column and affinity-purified essentially as described [20]. Fractions containing methylated CpG-rich Mse1 fragments were pooled and re-chromatographed before precipitation (Figure 3B). Purified DNA was resuspended in 1×TE and amplified in parallel with input DNA, using the GC Rich PCR system (Roche; 2 min at 95 °C; followed by 18 cycles of 95 °C for 1 min, T^ann^ °C for 1 min, 72 °C for 4 min; and a final extension of 72 °C for 7 min; universal primer_GGT CCA TCC AAC CGA TCT TA). MAP and Input DNAs (200 ng) were fluorescently labeled by random priming using the Bioprime labeling kit (Invitrogen), 1 × dNTS (10× dNTPS; 2 mM of each dATP, dGTP, dTTP, 1 mM dCTP) and 1.5 nmol of Cy3 or Cy5-dCTP (GE Healthcare). The labeled Input and MAP probes were purified (Invitrogen “Purelink”), pooled, and precipitated with 100 μg of human Cot-1 DNA (Invitrogen). Labeled DNA was resuspended in 400 μl of hybridisation buffer (2XSSC, 50% deionised formamide, 10 mM Tris-HCl [pH7.5], 10% dextran sulphate, 0.1% Tween 20), denatured at 100 °C for 10 min, snap-chilled on ice, and incubated for 1 h at 37 °C. The CGI microarrays were prehybridised with Cot-1 and herring sperm DNA (Sigma) before being hybridised for 48 h at 37 °C. Arrays were washed four times at 37 °C in 1× phosphate buffered saline/0.05% Tween 20, three times at 52 °C in 1 × saline sodium citrate, twice at RT in 1 × phosphate buffered saline/0.05% Tween 20, and finally rinsed in water, before being dried by centrifugation (500*g*).

### Microarray scanning and data analysis.

Arrays were scanned with a GenePix Autoloader 4200AL (Axon) and then processed using the GenePix Pro 6.0 (Axon) software package. All subsequent analysis was carried out with the LIMMA package in the *R* statistical environment. Features with poor signal-to-noise ratios were stabilised using a base value of 1,000 for background-subtracted intensities. Cy3 and Cy5 signals were transformed into *M* values (log_2_[red/green]) and normalised by print-tip loess. Each tissue analysis is represented by four microarrays comprising two independent replicates with respective dye swaps. Processed values were averaged through linear modeling and used to determine the relative enrichment of MAP DNA relative to Input. An *M* value of >1.5 was designated as the threshold for hypermethylation as determined by quantitative PCR (Figure 3C) and bisulfite genomic sequencing (Figures 3G and 3H, 4D–4G, and 5A and 5B). This threshold was confirmed as significant by calculation of a *t*-statistic by eBayes modeling and BH multiple testing correction. Differential methylation was deduced when features displayed an *M* value >1.5 in one or more tissues and a differential of 0.75 between tissues (upper boundary capped at *M* = 2.5). To avoid complications due to X chromosome inactivation, CGIs on sex chromosomes were not included in the analysis. In addition, spots that gave no signal on the microarray (NA values) and spots containing DNA in which CpG[o/e] values were <0.5 were excluded.

### Quantitative PCR.

Real-time PCR was carried out on MAP and Input material with iQ SYBR Green Supermix (Bio-Rad) on an iCycler (Bio-Rad) according to manufacturer's instructions. For primer sequences see Table S4.

### Bisulfite genomic sequencing.

Bisulfite treatment of genomic DNA was carried out as described by Feil et al. [55], and prepared for sequencing as outlined by Suzuki et al. [56]. Genomic DNA (5 μg) was digested by EcoRI prior to bisulfite treatment, and precipitated after the desulfonation step. Samples were resuspended in 1 ×Tris-EDTA buffer for subsequent PCR and sequencing reactions. Bisulfite specific primers were designed both manually and with the aid of the MethPrimer software [3] (sequences are available on request). PCR was carried out on the bisulfite-treated DNA using RedHot Taq DNA polymerase (Abgene) under the following conditions: 2 min at 94 °C; followed by 40 cycles of 94 °C for 50 s, T^ann^ °C for 50 s, 72 °C for 1 min; and a final extension of 72 °C for 5 min. PCR fragments were cloned using the Strataclone PCR cloning system (Stratagene) and at least ten products amplified (as above) and sequenced (BigDye Terminator v3.1 Cycle Sequencing Kit; Applied Biosystems). Methylation status and experimental quality control was carried out with the aid of BiqAnalyzer [57].

## Supporting Information

Dataset S1 Characterization of Human CGIs Identified as Being Methylated in One or More Somatic Tissues Included are sequence characteristics (G+C and CpG[o/e]), gene association (Gene ID, ENSEMBL nomenclature ), and the methylation profile in the four tissues tested (categories of methylation are colour-coded as for Figure 4B). The CpG island identifier (ID) corresponds to the arrayed CGI fragment. The genomic position of each CGI (Location) corresponds to the chromosomal coordinates derived from NCBI build 36.
(373 KB XLS)

Figure S1 Sequence Characteristics of CGIs Missed by NCBI-Strict NCBI-strict relies on base composition to identify CGIs, utilising threshold values for CpG[o/e] and G+C density (0.6% and 50%, respectively) as determinants. Boxplots of G+C and CpG[o/e] indicate that CGIs retained by the CXXC affinity matrix but missed by NCBI-strict have significantly reduced G+C base composition (*p*-value < 2.2e^−16^) and CpG[o/e] (*p*-value < 2.2e^−16^). A nonparametric distribution was determined using a Shapiro-Wilk test of normality and subsequent significance was determined using a two-sample Kolmogorov-Smirnov test (NCBI-missed *n* = 4,082 and all CGIs *n* = 13,305).
(48 KB DOC)

Figure S2 Sequence Properties of Methylated CGIs Bock and colleagues determined a number of DNA sequence features that are correlated with DNA methylation at CGIs [50]. Here we compare the sequence attributes of the methylated and total CGI sets with respect to DNA structure (stacking energy and base twist) and specific repeats (TGTG/CACA). Methylated CGIs show small but significant increase in stacking energy relative to all CGIs (*p*-value < 0.001). In contrast we found no significant difference in the base twist of methylated CGIs.
TGTG/CACA specific repeats were found to be significantly enriched in methylated CGIs (*p*-value < 0.001; see text for discussion). In contrast, all repetitive elements (as outlined in Repbase [58]) were found to be marginally depleted in methylated CGIs (*p*-value < 0.01, Wilcoxon rank sum test, *n* = 4,082 and 10,236). Stacking energy and base twist were calculated using the EMBOSS b-twisted program with default settings[59]. All distributions were tested for parametric distribution by the Shapiro-Wilk test of normality. Nonparametric significance values were determined using the Wilcoxon rank sum test (*n* = 4,082 and 10,236).
(66 KB DOC)

Table S1 Gene Association: CpG Islands Missed by NCBI-Strict All CGIs (*n* = 4,082) retained by the CXXC affinity matrix but not predicted by NCBI-strict were mapped relative to protein-coding genes. Gene overlap indicates the spatial association of CGIs relative to protein-coding genes.
(27 KB DOC)

Table S2 Methylated CGIs on Chromosome 16 and Chromosome X in Human Whole Blood DNA CGI arrays hybridised with MBD probe from male and female human blood DNA identify extensive methylation of X-linked CGIs. In contrast, the percentage of X-linked CGIs methylated on the single male X chromosome was comparable to the levels found on the human autosomes, as illustrated for Chr16.
(30 KB DOC)

Table S3 Developmental Gene Categories Are Associated with Differentially Methylated CpG Islands Ontology terms for gene-associated CGIs were compared with those for differentially methylated CGI-genes. Genes involved in developmental processes such a neurogenesis and segmentation are significantly enriched and include transcriptional regulators such as homeobox genes. Significantly enriched biological processes and molecular functions were determined using the Web-based Panther classification system (http://www.pantherdb.org/)[60].
(41 KB DOC)

Table S4 Quantitative PCR Primers for Microarray Validation CGI ID: CpG island identifiers which correspond to the CGI library.
*M* value: Log_2_[MBD/Input] for human blood microarray experiment.
(36 KB DOC)

## Abbreviations

CGI: CpG island
CAP: CXXC affinity purification
MAP: MBD affinity purification
TSS: transcription start site
