# Supplementary material for: A Novel CpG Island Set Identifies Tissue-Specific Methylation at Developmental Gene Loci
Source: PLoS Biol. 2008 Jan 29;6(1):e22. doi: 10.1371/journal.pbio.0060022 (PMC2214817; doi:10.1371/journal.pbio.0060022)
Supplement: Figure S1 — NCBI-strict relies on base composition to identify CGIs, utilising threshold values for CpG[o/e] and G+C density (0.6% and 50%, respectively) as determinants. Boxplots of G+C and CpG[o/e] indicate that CGIs retained by the CXXC affinity matrix but missed by NCBI-strict have significantly reduced G+C base composition (p-value < 2.2e−16) and CpG[o/e] (p-value < 2.2e−16). A nonparametric distribution was determined using a Shapiro-Wilk test of normality and subsequent significance was determined using a two-sample Kolmogorov-Smirnov test (NCBI-missed n = 4,082 and all CGIs n = 13,305). (48 KB DOC) [file pbio.0060022.sg001.doc]

**Figure S1 - Sequence characteristics of CGIs missed by NCBI strict**. The NCBI strict relies on base composition to identify CGIs, utilising threshold values for CpG[o/e] and G+C density (0.6 and 50% respectively) as determinants. Boxplots of G+C and CpG[o/e] indicate that islands retained by the CXXC affinity matrix but missed by NCBI strict have significantly reduced G+C base composition (p.value<2.2e-16*) and CpG[o/e] (p.value<2.2e-16*).


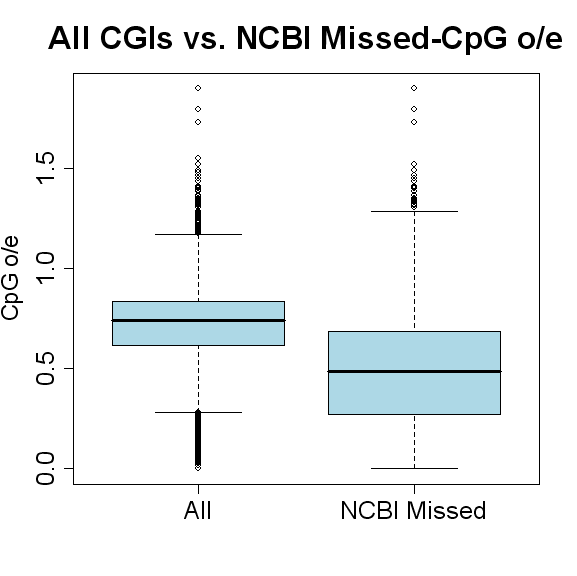

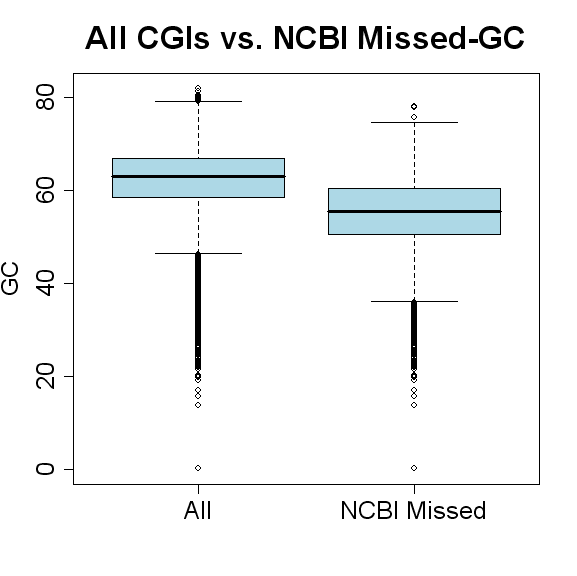


*A non-parametric distribution was determined using a Shapiro-Wilk test of normality and subsequent significance was determined using a two sample Kolmogorov-Smirnov tests. NCBI-missed and all CGIs (n=4082 and 13305 respectively).
