# Supplementary material for: A Novel CpG Island Set Identifies Tissue-Specific Methylation at Developmental Gene Loci
Source: PLoS Biol. 2008 Jan 29;6(1):e22. doi: 10.1371/journal.pbio.0060022 (PMC2214817; doi:10.1371/journal.pbio.0060022)
Supplement: Figure S2 — Bock and colleagues determined a number of DNA sequence features that are correlated with DNA methylation at CGIs [50]. Here we compare the sequence attributes of the methylated and total CGI sets with respect to DNA structure (stacking energy and base twist) and specific repeats (TGTG/CACA). Methylated CGIs show small but significant increase in stacking energy relative to all CGIs (p-value < 0.001). In contrast we found no significant difference in the base twist of methylated CGIs. TGTG/CACA specific repeats were found to be significantly enriched in methylated CGIs (p-value < 0.001; see text for discussion). In contrast, all repetitive elements (as outlined in Repbase [58]) were found to be marginally depleted in methylated CGIs (p-value < 0.01, Wilcoxon rank sum test, n = 4,082 and 10,236). Stacking energy and base twist were calculated using the EMBOSS b-twisted program with default settings[59]. All distributions were tested for parametric distribution by the Shapiro-Wilk test of normality. Nonparametric significance values were determined using the Wilcoxon rank sum test (n = 4,082 and 10,236). (66 KB DOC) [file pbio.0060022.sg002.doc]

**Fig S2 – Sequence properties of methylated CGIs.** Bock and colleagues determined a number of DNA sequence features which are correlated with DNA methylation at CGIs[1]. Here we compare the sequence attributes of the methylated and total CpG Island sets with respect to DNA structure (stacking energy and base twist) and specific repeats (TGTG/CACA). Methylated CpG islands show small but significant increase in stacking energy relative to all CGIs (p-value < 0.001). In contrast we found no significant difference in the base twist of methylated CGIs.


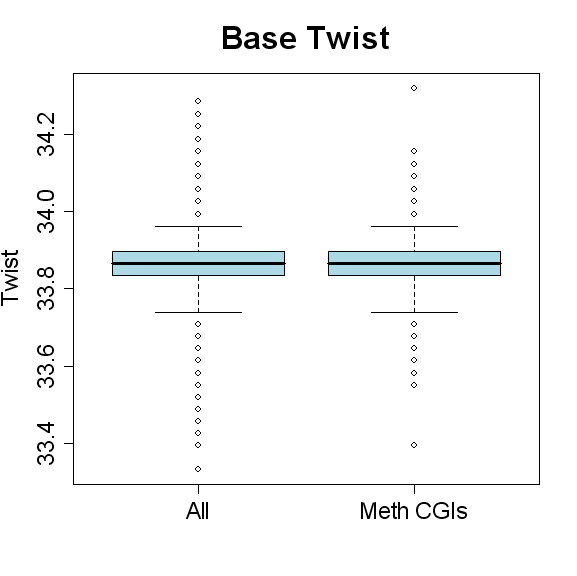

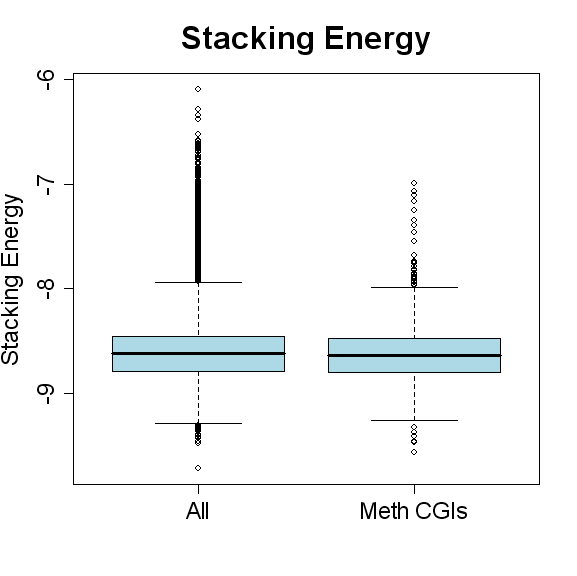


TGTG/CACA specific repeats were found to be significantly enriched in methylated CGIs (p-value < 0.001; see text for discussion). In contrast, all repetitive elements (as outlined in Repbase [2]) were found to be marginally depleted in methylated CGIs (p-value < 0.01, Wilcoxon rank sum test (n=4082 and 10236)


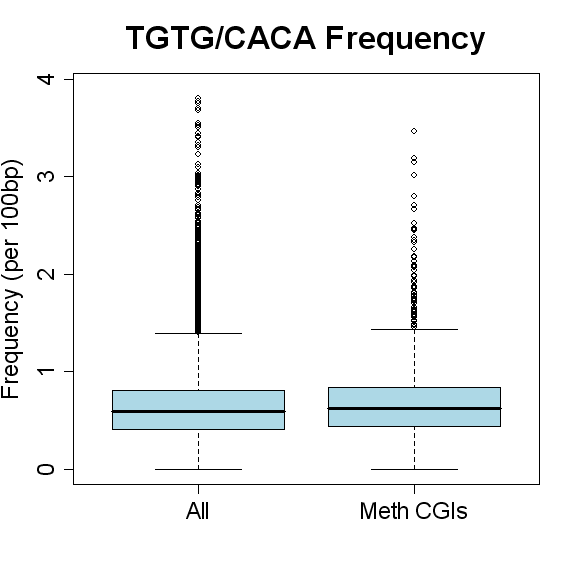


Stacking energy and base twist were calculated using the EMBOSS btwisted program with default settings[3]. All distributions were tested for parametric distribution by the the Shapiro-Wilk test of normality. Non parametric significance values were determined using the Wilcoxon rank sum test (n=4082 and 10236).

1. Bock C, Paulsen M, Tierling S, Mikeska T, Lengauer T, et al. (2006) CpG island methylation in human lymphocytes is highly correlated with DNA sequence, repeats, and predicted DNA structure. PLoS Genet 2: e26.

2. Jurka J, Kapitonov VV, Pavlicek A, Klonowski P, Kohany O, et al. (2005) Repbase Update, a database of eukaryotic repetitive elements. Cytogenet Genome Res 110: 462-467.

3. Rice P, Longden I, Bleasby A (2000) EMBOSS: the European Molecular Biology Open Software Suite. Trends Genet 16: 276-277.
