# Supplementary material for: A Novel CpG Island Set Identifies Tissue-Specific Methylation at Developmental Gene Loci
Source: PLoS Biol. 2008 Jan 29;6(1):e22. doi: 10.1371/journal.pbio.0060022 (PMC2214817; doi:10.1371/journal.pbio.0060022)
Supplement: Table S1 — All CGIs (n = 4,082) retained by the CXXC affinity matrix but not predicted by NCBI-strict were mapped relative to protein-coding genes. Gene overlap indicates the spatial association of CGIs relative to protein-coding genes. (27 KB DOC) [file pbio.0060022.st001.doc]

**Table S1 – Gene association: CpG islands missed by NCBI strict.**

All CGIs (n=4082) retained by the CXXC affinity matrix but not predicted by NCBI strict were mapped relative to protein coding genes. Gene overlap indicates the spatial association of CGIs relative to protein coding genes.

| **Gene overlap** | **Number of CGIs** | **Percentage of CGIs** |
| --- | --- | --- |
| 5’ | 778 | 19.1 |
| 3’ | 176 | 4.3 |
| Intragenic | 1421 | 34.8 |
| Intergenic | 1707 | 41.8 |
|  |  |  |
