# Supplementary material for: A Novel CpG Island Set Identifies Tissue-Specific Methylation at Developmental Gene Loci
Source: PLoS Biol. 2008 Jan 29;6(1):e22. doi: 10.1371/journal.pbio.0060022 (PMC2214817; doi:10.1371/journal.pbio.0060022)
Supplement: Table S2 — CGI arrays hybridised with MBD probe from male and female human blood DNA identify extensive methylation of X-linked CGIs. In contrast, the percentage of X-linked CGIs methylated on the single male X chromosome was comparable to the levels found on the human autosomes, as illustrated for Chr16. (30 KB DOC) [file pbio.0060022.st002.doc]

**Table S2 – Methylated CGIs on Chr16 and ChrX in Human Whole Blood**

**DNA**. CGI arrays hybridised with MBD probe from male and female human blood DNA identify extensive methylation of X-linked CGIs. In contrast, the percentage of X-linked CGIs methylated on the single male X chromosome were comparable to the levels found on the human autosomes, as illustrated for Chr16.

| **CpG Islands** | **Total** | **Methylated** | **Methylated (%)** |
| --- | --- | --- | --- |
| **Male Chr 16** | 569 | 49 | 8.6 |
| **Female Chr 16** | 569 | 62 | 10.9 |
| **Male X Chr** | 357 | 32 | 9.0 |
| **Female X Chr** | 357 | 151 | 42.3 |
