# Supplementary material for: A Novel CpG Island Set Identifies Tissue-Specific Methylation at Developmental Gene Loci
Source: PLoS Biol. 2008 Jan 29;6(1):e22. doi: 10.1371/journal.pbio.0060022 (PMC2214817; doi:10.1371/journal.pbio.0060022)
Supplement: Table S3 — Ontology terms for gene-associated CGIs were compared with those for differentially methylated CGI-genes. Genes involved in developmental processes such a neurogenesis and segmentation are significantly enriched and include transcriptional regulators such as homeobox genes. Significantly enriched biological processes and molecular functions were determined using the Web-based Panther classification system (http://www.pantherdb.org/)[60]. (41 KB DOC) [file pbio.0060022.st003.doc]

**Table S3: - Developmental gene categories are associated with differentially methylated CpG islands.** Ontology terms for gene associated CGIs were compared with those for differentially methylated CGI-genes. Genes involved in developmental processes such a neurogenesis and segmentation are significantly enriched and include transcriptional regulators such as homeobox genes. Significantly enriched biological processes and molecular functions were determined using the web based Panther classification system (<http://www.pantherdb.org/>)[1].

| **Biological Process** | **All genes* (n=9542)** | | **Methylated (observed n=490)** | **Methylated (expected)** | **p.value**** |
| --- | --- | --- | --- | --- | --- |
| Developmental processes | 1187 | | 112 | 60.95 | 4.00E-09 |
| mRNA transcription reg’n | 847 | | 85 | 43.5 | 4.61E-07 |
| Ectoderm development | 415 | | 51 | 21.31 | 1.94E-06 |
| mRNA transcription | 1093 | | 94 | 56.13 | 6.42E-05 |
| Neurogenesis | 383 | | 45 | 19.67 | 6.86E-05 |
| Segment specification | 71 | | 14 | 3.65 | 3.77E-03 |
| Mesoderm development | 326 | | 35 | 16.74 | 6.61E-03 |
|  |  | |  |  |  |
| **Molecular Function** | **All genes* (n=9542)** | | **Methylated (observed n=490)** | **Methylated (expected)** | **p.value**** |
| Homeobox transcription factor | 153 | | 37 | 7.86 | 3.16E-12 |
| Transcription factor | 1131 | | 99 | 58.08 | 2.54E-06 |
| Other DNA-binding protein | 180 | | 26 | 9.24 | 5.66E-04 |
|  | |  |  |  |  |

**CpG Island genes*

***Bonferroni correction for multiple testing.*

References:

1. Thomas PD, Campbell MJ, Kejariwal A, Mi H, Karlak B, et al. (2003) PANTHER: a library of protein families and subfamilies indexed by function. Genome Res 13: 2129-2141.
