# Supplementary material for: A Novel CpG Island Set Identifies Tissue-Specific Methylation at Developmental Gene Loci
Source: PLoS Biol. 2008 Jan 29;6(1):e22. doi: 10.1371/journal.pbio.0060022 (PMC2214817; doi:10.1371/journal.pbio.0060022)
Supplement: Table S4 — CGI ID: CpG island identifiers which correspond to the CGI library. M value: Log2[MBD/Input] for human blood microarray experiment. (36 KB DOC) [file pbio.0060022.st004.doc]

**Table S4 – Quantitative PCR primers for microarray validation.**

| **CGI ID** | **M value** | **Forward Primer** | **Reverse Primer** |
| --- | --- | --- | --- |
| I10484 | -1.72 | GGTTGTTTTCAAGATGGCGGA | CCTCAACTTCGAGCTAGCTC |
| I16200 | -0.82 | GCTAGAGATGGATGAGTCAC | GGGCAGTTCGGAATAAAAAC |
| I2206 | 0.06 | CGGTGAAAGAAGGAGGTGGG | CGTGACAAGAGAGTCCGCCT |
| I19422 | 0.65 | GGGTTTCTTCTATTCCTCGG | GGCCTGTCACCCATTTGTTC |
| I10295 | 1.3 | GGAACTTGGAGAGGAGGGCT | CCCAGCTCCTACTGTGGAAG |
| I8192 | 1.45 | CCTGGACCACACTCGTCCTA | GGACTTACTCCAGAAGGGCT |
| I6531 | 2.06 | CGTCTTCGGCAGGTACTCAG | GCAGCCCTGATGCTCAACTG |
| I11855 | 2.78 | GGCTAAAGAGCTGCTGCTGC | GGAGGCAGGTTCCTCTGTTG |
| I22248 | 3.48 | CGTTCCTCTCTGCACTCAGG | GCATCCGTGAGGCAGATGTC |
| I8257 | 3.99 | GCTCATTTCCACCGAGGTCAA | CGTAAGTGGGGACACTCATC |
| I19049 | 4.27 | CCATCCTCAGTTCCACCACC | GCAGGCCACGATAGATCAAC |
| I10840 | 5.1 | GGGTTTATTTTGGTGGGACG | CCTCAGTTTCTCCAGTCAAG |

CGI ID: CpG island identifiers which correspond to the CGI library.

M value: Log_2_[MBD/Input] for human blood microarray experiment.
